# Vertebroepidural metastasis of an ethmoid adenocarcinoma: A case report

**DOI:** 10.3892/ol.2013.1511

**Published:** 2013-08-02

**Authors:** MAGUETTE MBAYE, CLAUDIU POPA, FRANCESCO SIGNORELLI, NATHALIE STREICHENBERGER, ALAIN COSMIDIS, FABIO POZZI, JACQUES GUYOTAT

**Affiliations:** 1 Lyon Civil Hospitals, Pierre Wertheimer Neurological and Neurosurgical Hospital, Service of Neurosurgery D, Lyon 69500, France; 2 Magna Græcia University, Department of Experimental and Clinical Medicine, Catanzaro 88100, Italy; 3 Lyon Civil Hospitals, Pierre Wertheimer Neurological and Neurosurgical Hospital, Pathological Anatomy Service, Lyon 69500, France; 4 Croix Rousse Hospital, ENT Service, Lyon 69004, France

**Keywords:** ethmoid adenocarcinoma, vertebroepidural metastasis, spinal cord compression, surgical resection, complementary treatment

## Abstract

Ethmoid adenocarcinoma is the most frequent ethmoid tumor. To date, only a single case of spinal cord compression resulting from ethmoid adenocarcinoma has been reported. The current case study presents a recent case of vertebroepidural metastasis of an ethmoid adenocarcinoma leading to spinal cord compression. Modern imaging studies, including magnetic resonance imaging (MRI) and 18 fludeoxyglucose positron emission tomography (FDG PET), as well as histological and immunohistochemical analyses, have led to diagnoses of a metastasis of an ethmoid adenocarcinoma, which is a mucinous variant, dedifferentiated when compared to the primary tumor. The present case discusses current diagnostic and treatment protocols of this condition. Since survival rates associated with the primary tumor are improving, the incidence of spinal metastasis of ethmoid carcinomas is likely to increase in the future, therefore requiring appropriate diagnostic and therapeutic management.

## Introduction

Although rare, ethmoid adenocarcinoma is the most frequent ethmoid tumor. Chronic exposure to wood dust is a risk factor for its development (1,2). Progression is generally locoregional with invasion of adjacent structures, including the orbits, skull base, dura mater and brain. The prognosis of this malignancy is dependent on the extent of the disease at the time of diagnosis (3–6). Distant metastasis via the leptomeninges, metastasis to the neck nodes and hematogenous metastasis to distant organs have been reported (7–12). New neurological deficits due to metastasis are extremely rare and are usually an expression of leptomeningeal dissemination (10–16). The current case report presents a rare case of spinal cord compression due to vertebroepidural metastasis of an ethmoid adenocarcinoma. Written informed consent was obtained from the patient.

## Case report

### Clinical presentation and diagnosis of cancer

**Figure 1:**
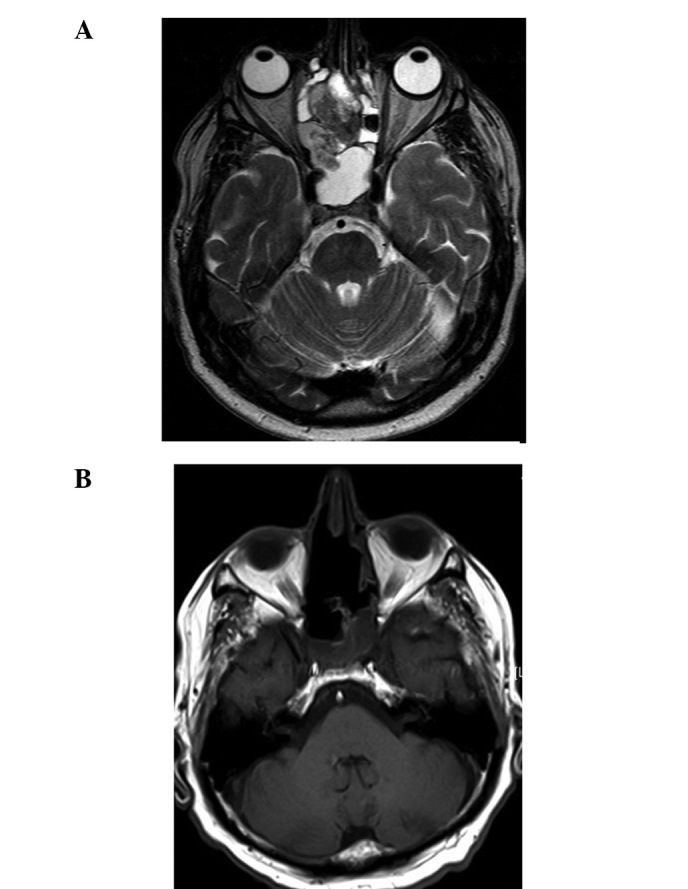
(A) Pre-operative axial T2-weighted head MRI revealing a right ethmoid adenocarcinoma (T4bN0M0 in Roux classification). (B) Post-operative axial gadolinium-enhanced T1-weighted head MRI following complete tumor removal.

A 70-year-old male, who had previously been a carpenter for 43 years, presented with a 4-month history of headaches with epistaxis. Enhanced head CT and MRI scans revealed a right ethmoid tumor with dural invasion (T4bN0M0 in Roux classification; Fig. 1A) (4,6). A transnasal biopsy confirmed the diagnosis of an adenocarcinoma, which was subsequently resected through a right paralateronasal approach by a multidisciplinary team, including a neurosurgeon and ENT surgeon (Fig. 1B). The histology of the primary lesion revealed an ethmoid adenocarcinoma, mucinous variant (Fig. 4A), expressing cytokeratin 20 (CK20) and partially expressing CK7.

### Treatment and clinical course

**Figure 2:**
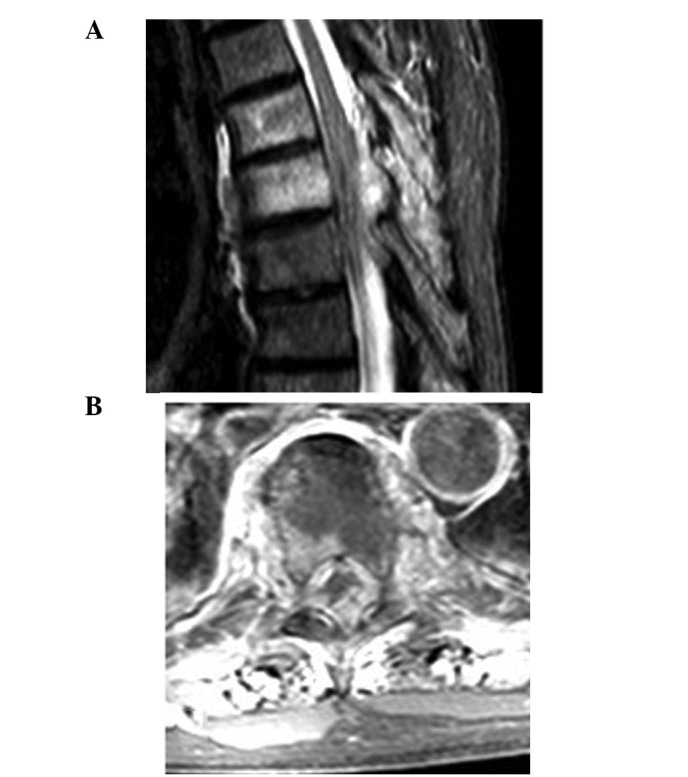
(A) Sagittal T2-weighted spinal MRI revealing metastatic infiltration of vertebral body bone marrow and spinous processes at T6 and T7, associated with metastatic epiduritis. (B) Axial gadolinium-enhanced T1-weighted spinal MRI at T6 demonstrating the epidural infiltration circumferentially compressing the spinal cord.

**Figure 3:**
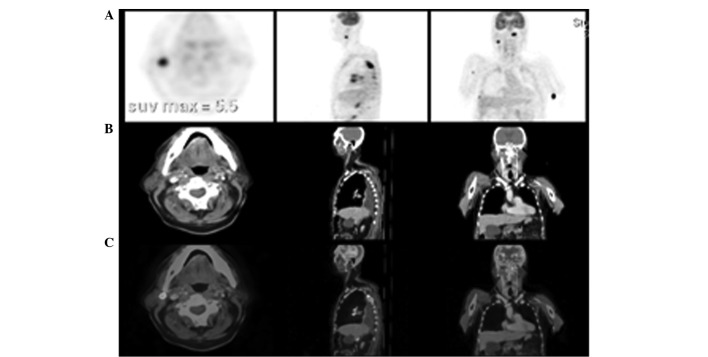
(A) ^18^F-fludeoxyglucose-positron emission tomography (^18^FDG-PET) and (B and C) fused PET-computed tomography (CT) revealing areas of hypermetabolism consistent with diffuse metastatic dissemination to the spine, right parotid gland, lungs and adrenal glands, as well as hilar, mediastinal and peritoneal lymph nodes. SUV, standardized uptake value.

**Figure 4:**
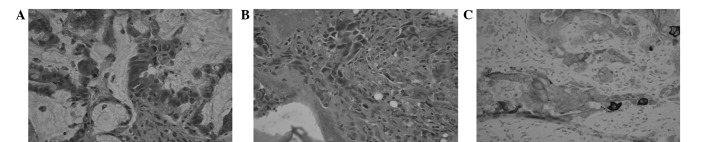
(A) Microscopic view of the primary tumor, stained with hematoxylin and eosin (magnification ×400), revealing a mucinous intestinal-type adenocarcinoma with small glands and solid islands floating in an abundant mucous substance. (B) Microscopic view of a histological specimen of the vertebroepidural metastasis demonstrating a mucinous intestinal-type adenocarcinoma formed by signet ring cells. The alveolar pattern, which exhibits a more differentiated appearance to the primary tumor, is lost. (C) Cytoplasmic CK20^+^/CK7^-^ immunostaining of the vertebroepidural metastasis (magnification, ×400).

The patient underwent post-operative intensity-modulated radiation therapy (60 Gy to the target volume). Two months later, the patient was admitted to the emergency department with an acute onset of the inability to empty the bladder. In addition, the patient reported severe dorsal pain and progressive lower limb weakness and numbness. A physical examination revealed unexplained weight loss and lower extremity weakness (3/5) to the point where the individual was no longer mobile. Proprioception and sensation to pain and temperature were diminished from the xiphoid process downward. Gadolinium-enhanced T1- and T2-weighted MRI scans revealed abnormal bone marrow signal enhancement at T5 and T6 (Fig. 2A) that was associated with an epidural mass that was encasing and severely compressing the spinal cord at T6-T8 (Fig. 2B). In addition, ^18^F-fludeoxyglucose-positron emission tomography (^18^FDG-PET) revealed diffuse metastatic dissemination to the spine, the hilar, mediastinal and peritoneal lymph nodes, the right parotid gland, the right gluteus, the lungs and each adrenal gland (Fig. 3). By contrast, there was no significant local hyperfixation, and a gadolinium-enhanced brain MRI excluded locoregional recurrence and dural or brain extension. A decompressive laminectomy and vertebroplasty were performed and post-operative standard beam external radiotherapy (48 Gy) was administered. This allowed for the partial remission of pain, but no sensorimotor improvement. Following 6 weeks of palliative and supportive care, the patient succumbed to his condition. Histological and immunohistochemical studies confirmed the diagnosis of epidural metastasis of an adenocarcinoma (Fig. 4B). Compared with the primary tumor, the metastatic lesion was dedifferentiated, demonstrating a partial expression of CK20 and no CK7 (Fig. 4C).

## Discussion

Although rare, ethmoid adenocarcinomas account for >50% of malignancies involving the ethmoid sinuses and ~15% of all sinonasal malignancies (2,7). Their progression is often locoregional with extension into the sinonasal cavities, orbit and eventually the intracranial compartment following infiltration of the dura mater. Meningeal extension (T4b in Roux classification) is associated with poor prognosis (3,4).

Distant metastases are identified in 6–30% of cases and are more often cerebromeningeal and osseous rather than visceral and ganglionic (1,3,4,8). Metastasis usually becomes evident between 13 and 19 months after the diagnosis of the primary tumor, although in certain cases metastases have been found as early as 2 months and as late as 42 months (3,9). Metastases may even occur in the absence of local relapse (3,8). Leptomeningeal carcinomatosis, with or without compressive epiduritis, has been reported in a number of case studies to be responsible for neurological impairment (9,10). The clinical outlook is variable and may include cauda equina syndrome (10,14), multiple involvement of the cranial and spinal nerves (3,11,12) and brain (8) or, extremely rarely, spinal cord dysfunction (13). The current study presents a case of spinal cord compression due to vertebroepidural metastasis of an ethmoid adenocarcinoma, which is, to the best of our knowledge, the second case described thus far (13). The present case is more recent and was examined pre-operatively by modern imaging studies, including MRI and ^18^FDG-PET, instead of myelography. In addition, histological and immunohistochemical analyses were performed, which are key tools for diagnosis. The histology of the primary lesion revealed an ethmoid adenocarcinoma, mucinous variant, that was expressing CK20 and partially expressing CK7. Compared with the primary tumor, the metastatic lesion was dedifferentiated, revealing a partial expression of CK20 and no CK7, which is not a rare observation (14). The expression of cytokeratins may indicate colonic, gastric or prostatic adenocarcinoma as a primary tumor, which more frequently leads to vertebroepidural metastasis rather than ethmoid adenocarcinomas (14). These observations indicated that an in-depth investigation was required for other forms of cancer, which were excluded in the present case by whole-body CT and PET scans and normal PSA levels.

Leptomeningeal seeding of ethmoid adenocarcinomas may be hematogenous, driven by cerebrospinal fluid (CSF) flow or occur through perivascular and perineural lymphatics (9,11,15). Seeding appears to occur more often when the tumor invades the intracranial compartment, as was consistent with observations of the current study and of previous cases involving the surgical opening of the dura mater (9,11,15). However, there is no clear correlation between tumor extension and the risk of leptomeningeal spread due to an insufficient number of studies on this area at present (3,8). Two mechanisms of invasion may be possible in the present case, namely via the CSF or the bloodstream. The short delay between the initial surgery and the diagnosis of vertebroepidural metastasis may support the first mechanism if the surgery had a favorable effect. However, metastasis may have already been present as pre-operative spinal MRI was not performed prior to the initial surgery. By contrast, multiple vertebral and systemic involvement supports the hematogenous mechanism.

These observations indicate the current issues associated with the initial management of patients affected by T4b tumors, and the rarity of cases explains the lack of available guidelines. Wen *et al* recommended a systemic search for tumoral cells within the CSF prior to radical surgery to exclude iatrogenic dissemination (7). However, spinal tap sensitivity is only 50% and increases to 90% if repeated twice (11). Gadolinium-enhanced spinal MRI is the most reliable diagnostic method for the identification of vertebroepidural metastasis, although it does not always reveal leptomeningeal dissemination (9). Despite the rarity of spinal leptomeningeal metastasis of ethmoid adenocarcinomas, the use of lumbar punctures and particularly gadolinium-enhanced spinal MRI, may be indicated prior to and following surgery in cases of T4b tumors. These procedures may also be recommended for more localized forms in cases of inadvertent intraoperative opening of the dura mater. Due to the possibility of late hematogenous dissemination, a quarterly gadolinium-enhanced spinal MRI would be suitable. However, at present, the prognosis of metastasis is extremely poor, particularly in cases of leptomeningeal dissemination, and survival times vary between 2 and 12 months following a diagnosis of secondary localization (1,4,7,16).

In conclusion, vertebroepidural metastasis of ethmoid carcinoma, although rare, must not be disregarded. Initial intradural extension may favor the onset of metastasis and justify a systematic search for neoplastic cells in the CSF and the performance of pre-operative and post-operative gadolinium-enhanced spinal MRI. Histological and immunohistochemical analyses are important for the differential diagnosis of more frequently occurring tumors and allow for establishment of a firm diagnosis. The prognosis of this type of metastasis is poor and depends more on the diffusion of the disease than on local recurrence. Due to current improvements in survival associated with the primary tumor, the incidence of spinal metastasis of ethmoid adenocarcinomas is likely to increase in frequency in the near future, therefore requiring appropriate diagnostic and therapeutic management.
